# Clinical Utility of a Cell-Free DNA Assay in Patients With Colorectal Cancer

**DOI:** 10.3389/fonc.2021.589673

**Published:** 2021-03-19

**Authors:** Ren-Hao Chan, Peng-Chan Lin, Shang-Hung Chen, Shao-Chieh Lin, Po-Chuan Chen, Bo-Wen Lin, Meng-Ru Shen, Yu-Min Yeh

**Affiliations:** ^1^Division of Colorectal Surgery, Department of Surgery, National Cheng Kung University Hospital, College of Medicine, National Cheng Kung University, Tainan, Taiwan; ^2^Department of Internal Medicine, National Cheng Kung University Hospital, College of Medicine, National Cheng Kung University, Tainan, Taiwan; ^3^Department of Obstetrics and Gynecology, National Cheng Kung University Hospital, College of Medicine, National Cheng Kung University, Tainan, Taiwan

**Keywords:** cfDNA, colorectal cancer, TP53, KRAS, BRAF, ERBB2 amplification

## Abstract

The analysis of cell-free DNA (cfDNA) is rapidly emerging as a powerful approach to guide the clinical care of cancer patients. Several comprehensive cfDNA assays designed to detect mutations across several genes are now available. Here, we analyzed the use of a cfDNA panel in colorectal cancer (CRC) patients. Twenty-eight CRC patients with relapse or metastatic disease and 31 patients with no evidence of disease (NED) were enrolled. Genomic alterations in cfDNA were analyzed by the Oncomine™ Pan-Cancer Cell-Free Assay that detects hotspot mutations, small indels, copy number changes, and gene fusions across 52 genes. In the NED group, genomic alterations in cfDNA were detected in 12/31 patients (38.7%). The detection of alterations was more common in patients who were ≥60 years old, and the most common genomic alteration was a *TP53* mutation. Fifty percent of the *TP53* mutations were frequently or very frequently found in human cancers. Among 28 patients with relapse or metastatic disease, 22 (78.6%) had genomic alterations in cfDNA. The alterations were detected most frequently in *TP53* (*n* = 10), followed by *KRAS* (*n* = 9). Actionable targets for CRC, including *ERBB2* amplification and *BRAF* mutations, could be identified by this cfDNA assay. Compared with mutational profiling routinely analyzed using tumor samples, several additional targets with currently available therapies, including *IDH1, IDH2*, and *PDGFRA* mutations, were discovered. The cfDNA assay could identify potentially actionable targets for CRC. Identifying how to filter out cancer-like genomic alterations not derived from tumors remains a challenge.

## Introduction

Colorectal cancer (CRC) is still a major leading cause of cancer-related death worldwide. There are about 1.2 million new cases of CRC diagnosed each year, with 900,000 deaths attributed to the disease (1). Patients with stage I CRC have an excellent outcome. Specifically, the 5-years survival rate after surgical resection alone is around 90%. Postoperative adjuvant chemotherapy with 5-fluorouracil, leucovorin, and oxaliplatin has been demonstrated to reduce the risk of recurrence and improve overall survival in patients with stage II and III CRC (2). However, recurrence still develops in ~30% of patients, and the side effects of chemotherapy significantly impair the patients' quality of life, especially oxaliplatin-related neurotoxicity. The biggest challenge in an adjuvant setting is to correctly distinguish between patients who have residual disease that needs more aggressive postoperative adjuvant therapy and those who are cured of the disease, in which observation only is enough to avoid unnecessary treatment-related toxicities. In the context of metastatic CRC, the current treatment involves active chemotherapeutic drugs in combination with targeted agents (3). Despite the promising therapeutic efficacy, disease progression inevitably develops in the majority of patients. Identifying the emergence of resistant mutations and detecting the actionable targets to guide subsequent therapeutic strategies are still important issues during the treatment of metastatic CRC (4). However, a re-biopsy to obtain tumor tissue may not be always available, and tumor heterogeneity might limit the detection of resistance alterations in a single tumor biopsy (4, 5). Therefore, the development of a useful tool that could detect the residual disease, identify actionable targets, and monitor the emergence of resistance is still an unmet clinical need for the care of patients with metastatic CRC.

Circulating cell-free DNAs (cfDNA) are double-stranded DNA fragments that can be detected in the non-cellular component of blood (6). In patients with cancer, cfDNA is shed from both normal and cancer cells. Elevated cfDNA concentrations have been observed in cancer patients with cancer (7) and the cfDNA integrity, calculated as the ratio of short to long DNA fragment concentration, has also been reported to be a potential diagnostic marker of cancer (8). Analyzing tumor-derived cfDNA (ctDNA) has emerged as a promising strategy for the care of CRC patients. Several studies demonstrated ctDNA could be used to monitor residual disease in CRC patients after surgery (9–12). Positive ctDNA results, suggesting the presence of residual disease, were significantly associated with a higher risk of recurrence. In the metastatic setting, recent studies showed ctDNA could be used to track clonal evolution and identify primary or acquired resistance during treatment with an anti-epidermal growth factor receptor (EGFR) monoclonal antibody (13). Moreover, the mutational profiling of CRC patients could be performed by analyzing ctDNA, and the results were comparable to those generated from direct tumor sequencing (14). Therefore, the analysis of ctDNA appears to be a reliable approach to improve the clinical management of CRC patients who are undergoing surgery or chemotherapy.

Currently, several commercially available tests designed to detect different types of mutations across a wide range of genes can be used to identify genomic alterations in the cfDNA of cancer patients. The detection of mutations identified in tumor tissues and/or applying samples of reference DNA with pre-specified dilutions were often used to establish the analytical validity (15). However, the data of clinical validity and utility when applying commercial cfDNA tests in CRC patients in different clinical scenario were limited. Few studies showed sequencing by commercial cfDNA assays could provide timely mutational information comparable to those analyzed by direct tumor sequencing (14, 16, 17). The clinical applications of these commercial cfDNA assays in CRC patients after surgery are seldom reported. The main question we asked in this study is the clinical utility of a commercial cfDNA in CRC patients. Here, we enrolled 59 CRC patients, including 28 and 31 patients with and without clinical evidence of disease, to test the utility of Oncomine™ Pan-Cancer Cell-Free Assay, a commercial cfDNA assay, in monitoring residual disease and assessing molecular profiles. Genomic alterations in cfDNA were detected in 38.7% of patients with no clinical evidence of disease, and the detection of alterations was more common in older patients. The most common alteration detected in patients without evidence of disease was a tumor protein p53 (*TP53*) mutation, and 50% of the *TP53* mutations were frequently or very frequently found in human cancers. In terms of actionable targets, common druggable mutations for CRC, including proto-oncogene B-Raf (*BRAF*) mutations and receptor tyrosine-protein erbB-2 kinase (*ERBB2*) amplification, could be detected using this commercial cfDNA assay. Several potential targets with currently available therapies, including isocitrate dehydrogenase 1 (*IDH1*), *IDH2*, and platelet-derived growth factor receptor A (*PDGFRA*) mutations, were discovered, but the benefits of targeting these mutations in CRC still require further investigation.

## Materials and Methods

### Study Cohort

Patients with histologically confirmed CRC from National Cheng Kung University Hospital (NCKUH) were prospectively enrolled. Study patients were categorized into two groups depending on whether their most recent image studies showed the presence of disease (relapse or metastasis group) or no evidence of disease (NED group). Patients in the NED group received the radical resection of the CRC between 2000 and 2018. The clinical characteristics, including age, gender, tumor histology, site of the primary tumor, mutation status of Kirsten rat sarcoma 2 viral oncogene homolog (*KRAS)*, neuroblastoma RAS viral oncogene homolog (*NRAS*), *BRAF*, and human epidermal growth factor receptor 2 (*HER2*) genes, and mismatch repair/microsatellite instability (MMR/MSI) status were obtained from medical records. Tumor tissues, either the primary or metastatic tumors, were used for mutational analysis of these genes. After enrollment, 10 mL of peripheral blood were collected for targeted next-generation sequencing of cfDNA during routine follow-up at outpatient clinic. This study was approved by the institutional review board of NCKUH (A-ER-108-033). All participants provided written informed consent before enrollment.

### Sample Collection, Genomic DNA, and cfDNA Extraction

Ten milliliters of peripheral blood were collected in a PAXgene Blood ccfDNA tube and shipped to the lab at room temperature (15–25°C). To isolate plasma, the blood sample was centrifuged at 1,900 × g for 10 min and the plasma was transferred to a 2.0 mL microcentrifuge tube. After centrifugation at room temperature for 10 min at 12,000 × g, the cfDNA was extracted using the Applied Biosystems™ MagMAX™ Cell-Free DNA Isolation Kit. The concentration of cfDNA was measured by a Qubit™ Fluorometer 3.0 using Qubit™ dsDNA and RNA High Sensitivity Assays (Thermo Fisher Scientific). The gDNA was extracted using a QIAamp DNA Blood Mini Kit. The concentration of gDNA was detected by an Invitrogen™ Qubit™ Fluorometer using the Qubit™ dsDNA High Sensitivity Assay. Details of the DNA input in the NED and relapse or metastasis group were shown in Supplementary Table 1.

### Library Preparation and Sequencing

Genomic alterations in cfDNA were analyzed by the Oncomine™ Pan-Cancer Cell-Free assay that is designed to detect single-nucleotide variants (SNV), insertions/deletions (indel), copy number variants (CNV), and gene fusions across 52 genes. Target regions from cfDNA were amplified using the Oncomine™ cfDNA Assay (Thermo Fisher Scientific). Library construction of the amplicons was performed according to the Oncomine cfDNA Assays Part I: Library Preparation or Oncomine Cell-Free Research Assay (Thermo Fisher Scientific) User Guide. Template preparation and chip loading were conducted with the Ion 530 Kit-Chef (Thermo Fisher Scientific). The Ion 530 Kit-Chef was used with the Ion S5 XL sequencer (Thermo Fisher Scientific), as described in the Ion 530 Kit-Chef User Guide.

### Bioinformatics and Statistical Analysis

Data quality control, alignment, variant calling, and limit of detection (LOD) calculations were conducted using a locked data analysis pipeline, Torrent Suite version 5.10 (Thermo Fisher Scientific), and Ion Reporter version 5.10 (Thermo Fisher Scientific). This analysis pipeline provided SNV and indel callings with allelic frequencies as low as 0.05%, as well as a gene fusion calling with the sequencing read counts of ≥25 reads. For CNV analysis, copy numbers with >4.0 or <1.5 N in the assayed sample were considered copy number variations. The reference genome was hg19. Variant annotation was performed using Annovar version 2018Apr16. Variants in vcf files were retained if they satisfied one of the following criteria: (1) allele frequency > LOD, or (2) pathogenic, likely pathogenic, or drug response assigned by ClinVar (version 2019Mar05). The test was reported as positive if any variant was observed.

### Databases for the Mutational Analysis of the *TP53* Gene

Whole-genome sequencing data from 499 normal Taiwanese subjects provided by the Taiwan BioBank were used to analyze germline *TP53* genetic variants in a normal population (18). The distribution and frequency of germline *TP53* variants were compared with the *TP53* mutations identified in the cfDNA of CRC patients. To further analyze the mutational information, the detected *TP53* mutations in cfDNA were queried in Seshat, which is an online tool for the analysis of *TP53* mutations based on the Universal Mutation Database (UMD) TP53 database (19).

### Statistical Analysis

Disease-free survival was defined as the time from curative surgery to the collection of blood samples for cfDNA analysis. An unpaired *t*-test was used to compare the mean ages of CRC patients with positive and negative cfDNA analyses. Fisher's exact test was used to analyze the relationship between the results of cfDNA analysis and age, gender, and the location of the primary tumor. All the statistical analyses were performed using GraphPad Prism 9 software.

## Results

### Clinical Characteristics of the CRC Cohort

A total of 59 CRC patients, including 31 patients in the NED group and 28 in the relapse or metastasis group, were enrolled in this study. The clinical characteristics of the patients are shown in Table 1 and Supplementary Table 1. The median age of the patients in the NED group was 63 years, 58.1% of the patients were male, and 77.4% had a primary tumor in their left colon. Distant metastasis developed in one of the seven patients with stage II disease and five of 23 patients with stage III disease after the initial surgery. All of these six patients with recurrent disease and one patient who had stage IV CRC at the initial diagnosis received surgical resection of the metastatic lesions with curative intent. Recent image studies of these 31 patients before the collection of cfDNA samples showed NED. The median disease-free survival in this group was 4.1 years, and 20 (65%) of the 31 patients had been disease-free for more than 3 years. Among patients with available mutational profiles, *KRAS* and *BRAF* V600E mutations were detected in 50 and 25% of the patients, respectively, without any *NRAS* mutation, deficient MMR/MSI, or *HER2* amplification. In the relapse or metastasis group, the median age was 54 years, 60.7% of the patients were male, and 78.6% had left colon cancer. The percentage of patients with *KRAS, NRAS*, and *BRAF* V600E mutations were 42.9, 0, and 10.7%, respectively. One patient (3.6%) had deficient MMR/MSI CRC, and 1 patient (3.7%) had *HER2*-positive CRC.

**Table 1:** Clinical characteristics.

**Clinical characteristic**	**NED (*n =* 31)**	**Relapse or metastasis (*n =* 28)**	***p*-value**
**Age**			
Median, years (range)	63 (36~80)	54 (35~81)	0.1165
**Gender**			
Male, No. (%)	18 (58.1)	17 (60.7)	>0.9999
Female, No. (%)	13 (41.9)	11 (39.3)	
**Histology**			
Adenocarcinoma, No. (%)	31 (100)	28 (100)	>0.9999
**Site of the primary tumor**			
Right, No. (%)	7 (22.6)	6 (21.4)	>0.9999
Left, No. (%)	24 (77.4)	22 (78.6)	
***KRAS***			
Wild type, No. (%)	10 (50.0)	16 (57.1)	0.7704
Mutant, No. (%)	10 (50.0)	12 (42.9)	
G12D, No. (%)	4 (20.0)	3 (10.7)	
G13D, No. (%)	3 (15.0)	3 (10.7)	
G12V, No. (%)	2 (10.0)	1 (3.6)	
Others, No. (%)	1 (5.0)	5 (17.9)	
Not available, No.	11	0	
***NRAS***			
Wild type, No. (%)	15 (100)	28 (100)	>0.9999
Mutant, No. (%)	0 (0)	0 (0)	
Not available	16	0	
***BRAF*** **V600E**			
Wild type, No. (%)	3 (75.0)	25 (89.3)	0.4306
Mutant, No. (%)	1 (25.0)	3 (10.7)	
Not available, No.	27	0	
**MMR/MSI status**			
Proficient, No. (%)	(100)	27 (96.4)	>0.9999
Deficient, No. (%)	0 (0)	1 (3.6)	
Not available, No.	14	0	
***HER2*** **status**			
Positive, No. (%)	0 (0)	1 (3.7)	>0.9999
Negative, No. (%)	3 (100)	26 (96.3)	
Not available, No.	28	1	

*HER2, human epidermal growth factor receptor 2; MMR, mismatch repair; MSI, microsatellite insufficiency; NED, no clinical evidence of disease*.

### Genomic Alterations in the Relapse or Metastasis Group

**Figure 1:**
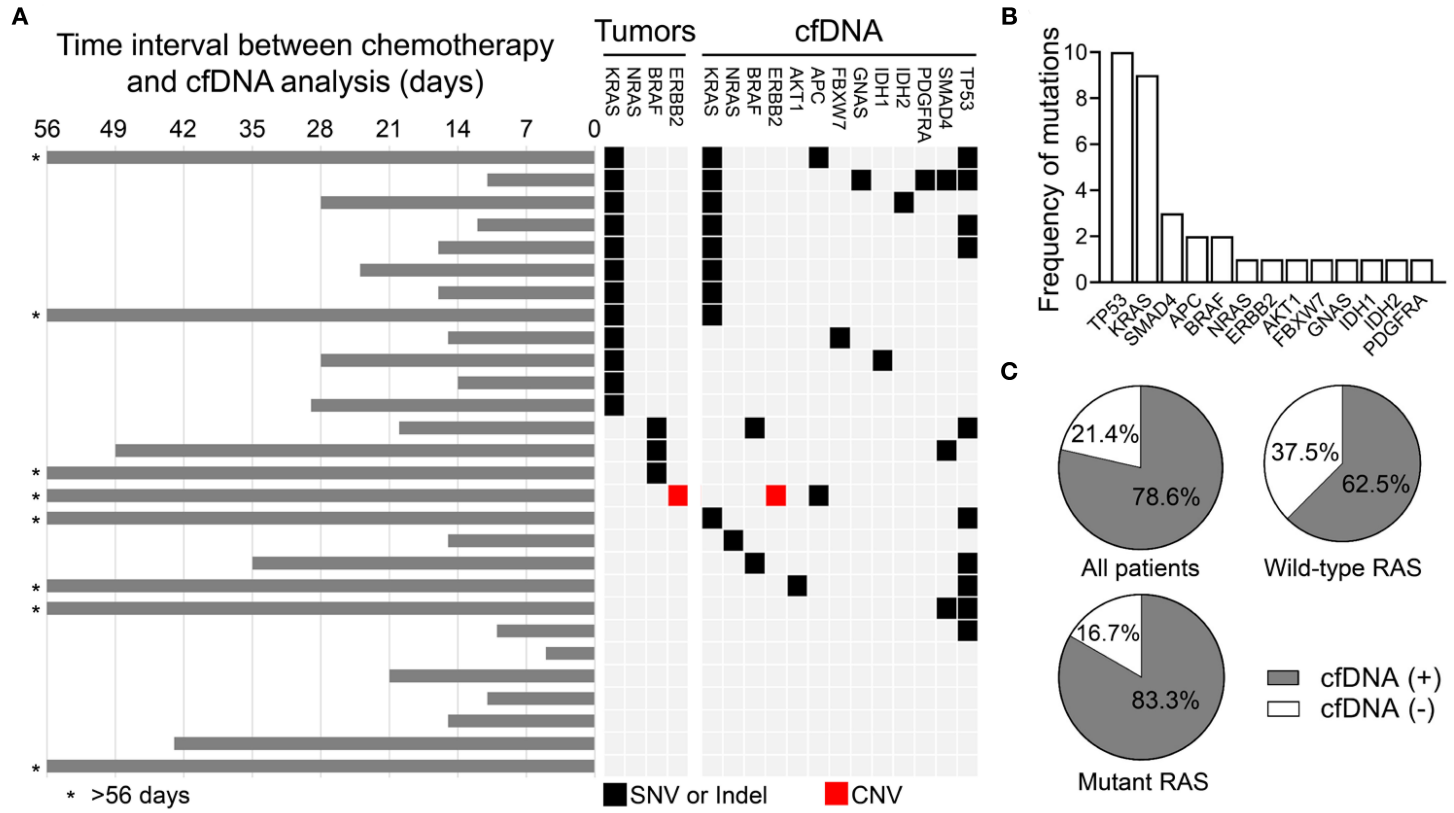
Genomic alterations detected in the cfDNA analysis in the relapse or metastasis group. **(A)** Schematic showing the genomic alterations detected in cfDNA and the time interval between the end of chemotherapy and the cfDNA analysis. **(B)** Frequency of detected genomic alterations. **(C)** Percentage of all patients, RAS wild type patients, and RAS mutant patients with and without detected genomic alterations.

In the relapse or metastasis group, genomic alterations in cfDNA were detected in 22 of 28 patients (78.6%). The time interval between chemotherapy and cfDNA analysis, the mutational profiling routinely analyzed using tumor samples, and genomic alterations detected in the cfDNA are shown in Figure 1A. The alterations were detected most frequently in *TP53* (*n* = 10), followed by *KRAS* (*n* = 9), *SMAD4* (*n* = 3), *APC* (*n* = 2), *BRAF* (*n* = 2), *NRAS* (*n* = 1), *ERBB2* (*n* = 1), *AKT1* (*n* = 1), *FBXW7* (*n* = 1), *GNAS* (*n* = 1), *IDH1* (*n* = 1), *IDH2* (*n* = 1), and *PDGFRA* (*n* = 1), as shown in Figure 1B and Supplementary Table 2. For 12 patients with *KRAS* mutant CRC, 10 (83.3%) had positive cfDNA analysis (Figure 1C), and the same *KRAS* mutation was detected in the cfDNA of eight patients (66.7%; Figure 1A). Among patients with wild type *KRAS*, genomic alterations in cfDNA were only detected in 10 of 16 (62.5%) patients. *RAS* mutations in cfDNA were detected in two of the 10 patients, including one *KRAS* Q61H and one *NRAS* Q61H mutation. Disease progression in patients on anti-EGFR therapy developed in these two patients about 6–11 months prior to the collection of blood samples for cfDNA analysis. Three patients had *BRAF*-mutant CRC, but a *BRAF* alteration in cfDNA was only detected in one of them. The Oncomine™ Pan-Cancer Cell-Free Assay was designed to detect multiple tumor-derived genetic alterations, including SNVs, short indels, gene fusions, and CNVs. Based on the diagnostic criteria of the HERACLES trial, a phase II study investigating the dual HER2 blockade in patients with HER2-amplified metastatic CRC (20), one of the 28 patients in the relapse or metastasis group had HER2-positive CRC and HER2 amplification was also detected in the cfDNA of the same patient. Compared with mutational profiling routinely analyzed using tumor samples, several additional genomic alterations with currently available therapies were discovered, including *IDH1, IDH2*, and *PDGFRA* mutations.

### Genomic Alterations in the NED Group

**Figure 2:**
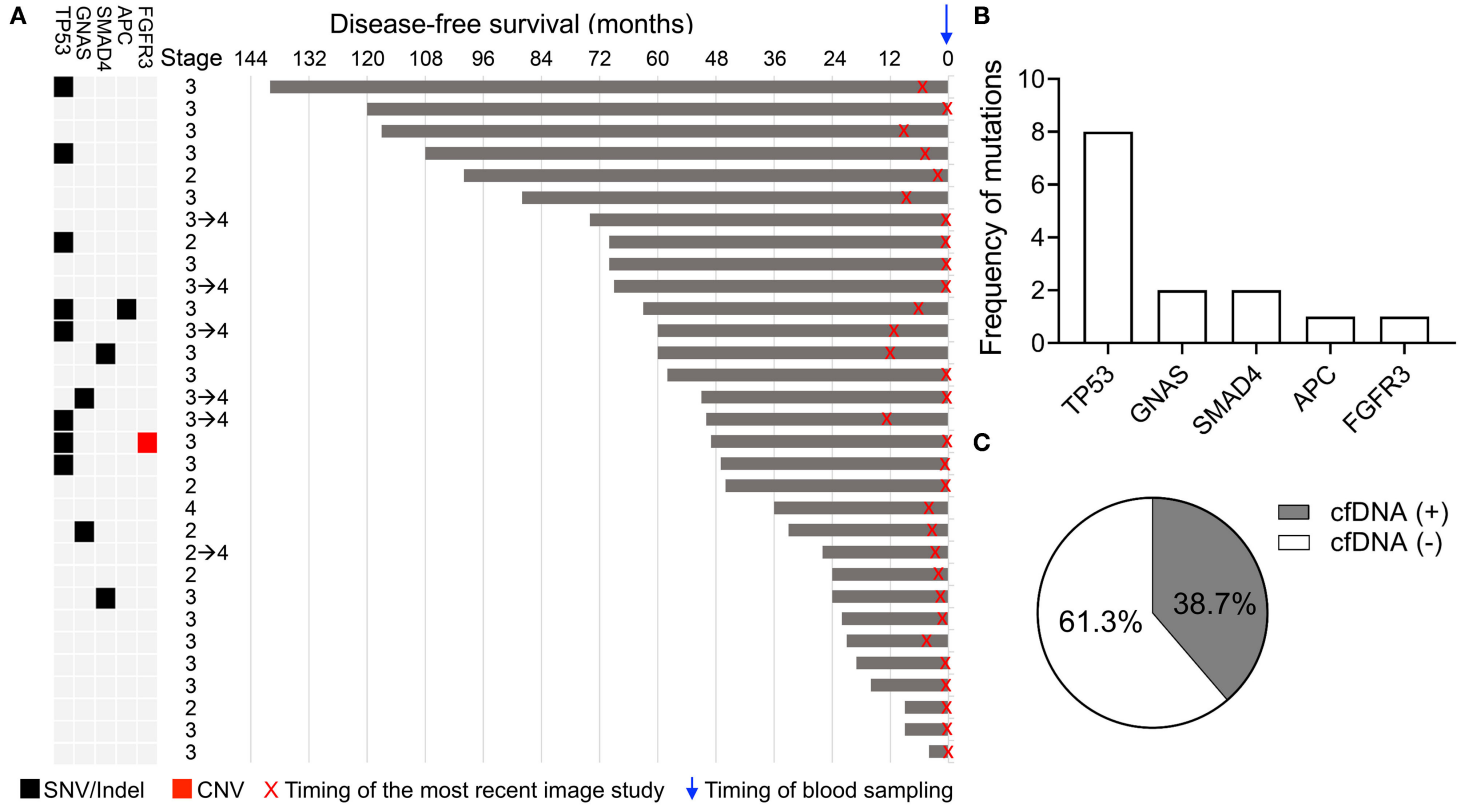
Genomic alterations detected in the cfDNA analysis in the NED group. **(A)** Schematic showing the clinical stage, duration of disease-free survival, and alterations detected in cfDNA. X indicates the timing of the most recent imaging study and the blue arrow indicates the timing of blood sampling. **(B)** Frequency of genomic alterations. **(C)** Percentage of patients with and without detected genomic alterations.

In the NED group, genomic alterations in cfDNA were detected in 12 of 31 (38.7%) patients. Detected alterations among the 12 cfDNA-positive samples included eight mutations in *TP53*, two mutations in *GNAS*, two mutations in *SMAD4*, one mutation in *APC*, and one deletion in *FGFR3* (Figures 2A,B). In one patient, variants were detected in both *TP53* and *APC* genes, and one patient had both a *TP53* mutation and *FGFR3* deletion. The details of the mutations detected in cfDNA are shown in Supplementary Table 3. The remaining 19 patients (61.3%) had no identified alteration in the cfDNA analysis (Figure 2C). A total of 15 and 35 mutations were detected in the NED and relapse or metastasis group, respectively (Supplementary Tables 2, 3). The mean of the mutant frequency in the NED group was lower than that in the relapse or metastasis group (*p* = 0.0523, Supplementary Figure 1). Considering the results from both the relapse or metastasis group and the NED group, the sensitivity and specificity of using the results of cfDNA analysis to determine the presence or absence of disease was 78.6 and 61.3%, respectively.

### The Clinical Characteristics and cfDNA Findings in the NED Group

**Figure 3:**
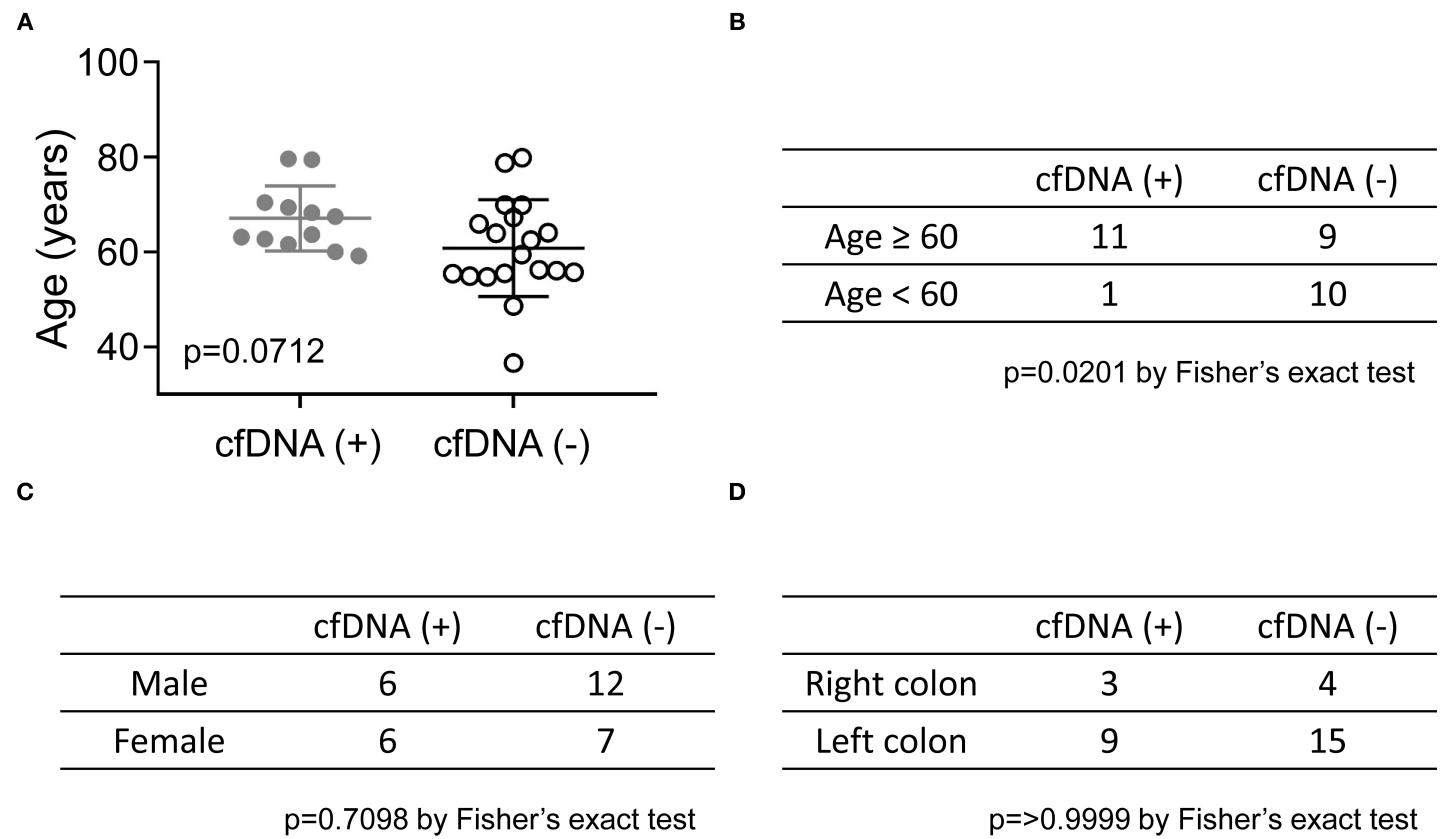
The correlation between the results of cfDNA analysis and age, gender, and the location of the primary tumor in the NED group. **(A)** The mean ages of CRC patients with positive and negative cfDNA analyses are shown and compared by an unpaired *t*-test. The Fisher's exact test was used to analyze the relationship between the results of the cfDNA analysis and age **(B)**, gender **(C)**, and site of the primary tumor **(D)**.

Although cfDNA analysis was considered a useful molecular tool for the early detection of relapse, the positive cfDNA findings in 12 patients, suggesting the presence of residual disease, were not consistent with their clinical status. Recent imaging studies of these patients all showed NED, and 10 of the 12 patients had been clinically disease-free for more than 3 years after curative surgery (Figure 2A). To identify the potential clinical features associated with the positive cfDNA test in the NED group, the correlation between the clinical characteristics and results of cfDNA test were analyzed. In the NED group, the mean age of patients with and without genomic alterations detected in cfDNA was 67.1 and 60.8 years, respectively (Figure 3A). Among 20 patients who were 60 or older, 11 (55%) showed positive cfDNA analysis. In contrast, genomic alterations in cfDNA were only detected in one of 11 (9.1%) patients who were younger than 60. Therefore, positive cfDNA analysis was more common in patients aged 60 or older (*p* = 0.0201; Figure 3B). Other clinical characteristics, including gender and the location of the primary tumor, were not associated with the result of cfDNA analysis (Figures 3C,D).

### *TP53* Mutations in the NED Group

**Figure 4:**
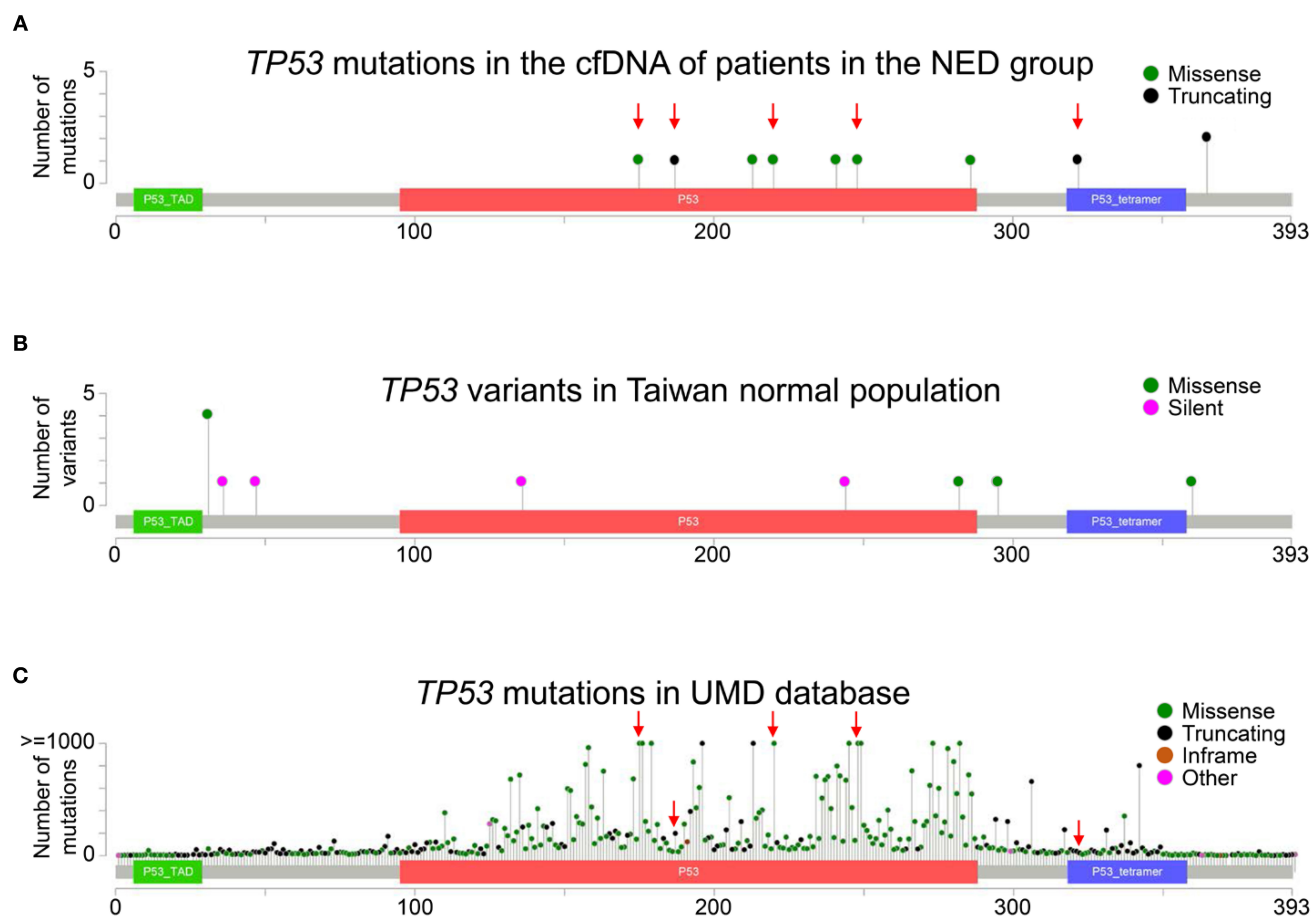
Distribution of *TP53* mutations. **(A)** The distribution of *TP53* mutations in the cfDNA analysis in the NED group is shown. The red arrows indicate the mutations that were “frequently” or “very frequently” found in the UMD TP53 database. **(B)** The whole-genome data of 499 normal Taiwanese subjects from the Taiwan BioBank were used to analyze the distribution of germline *TP53* genetic variants in a normal population. **(C)** The frequency and distribution of *TP53* mutations in all cancers in the UMD TP53 database are shown. The red arrows indicate mutations “frequently” or “very frequently” detected in the UMD database that were also detected in the NED group. Truncating mutations included non-sense, non-stop, frameshift deletion, frameshift insertion, and splice site mutations. Inframe mutations included inframe deletions and inframe insertions. Other mutations included all other types of mutations.

Among genomic alterations detected in the NED group, the most common alteration was a *TP53* mutation. The *TP53* gene is located on chromosome 17 (17p13.1) and consists of 12 exons. The Pan-Cancer Cell-Free assay was designed to detect mutations in exons 2–11 of the *TP53* gene. Ten different *TP53* mutations were detected in 8 patients in the NED group (Figure 4A), and these *TP53* mutations were clustered in exons 5–10. The details of these *TP53* mutations are shown in Supplementary Table 2. When analyzing the germline genetic variants of the *TP53* gene from the Taiwan BioBank, the result showed that the *TP53* mutations detected in cfDNA were different from the common germline variants in the normal population (Figure 4B). In contrast, five of the ten *TP53* mutations were “frequently” or “very frequently” identified in human cancers when these mutations were queried in Seshat, an online tool for the analysis of *TP53* mutations based on the UMD TP53 database (Figure 4C and Supplementary Table 4). These results suggest that the *TP53* mutations detected in cfDNA were the common clonal *TP53* mutations found in human cancers instead of the germline genetic variants.

## Discussion

cfDNA from plasma is becoming a useful tool in the clinical care of cancer patients. Several commercial cfDNA tests designed to detect different types of mutations across a wide range of genes are currently available. Here, we demonstrated the utility of the Oncomine™ Pan-Cancer Cell-free Assay in CRC patients with and without clinical evidence of disease. When using the results of cfDNA analysis to determine the presence or absence of disease, our study showed the sensitivity and specificity of this cfDNA assay were 78.6 and 61.3%, respectively. This cfDNA assay could identify important actionable mutations in CRC, such as *BRAF* mutations and *ERBB2* amplification. In addition, several potential targets with currently available therapies, including *IDH1, IDH2*, and *PDGFRA* mutations, were discovered. Positive cfDNA analysis was also observed in 38.7% of patients without clinical evidence of disease, and the detection of alterations was more common in patients who were 60 years or older. The most common alteration was a *TP53* mutation, and 50% of the *TP53* mutations were common *TP53* mutations found in human cancers.

The detection of residual disease is an important application of cfDNA analysis. For CRC patients, several prior studies selected the patient-specific somatic mutations identified in tumor tissues for cfDNA analysis, and the results showed the detection of alterations in cfDNA was associated with a higher risk of relapse in patients with stages I to III of the disease (10, 11). The specificity of personalized cfDNA analysis was very high (96–100%), whereas the sensitivity in predicting relapse was only ~40–50%. In this study, the Oncomine™ Pan-Cancer Cell-Free Assay, a commercially available 52-gene cfDNA panel, was used in CRC patients with and without clinical evidence of disease. When the detection of alterations in cfDNA was performed to determine the presence of disease, the sensitivity and specificity of this cfDNA assay were 78.6 and 61.3%, respectively. These results suggest that the use of the commercial comprehensive cfDNA assay in an adjuvant setting might have the potential to provide better sensitivity than personalized cfDNA analysis. The simultaneous analysis of mutations across 52 different genes may contribute to the increased sensitivity. However, the lack of specificity is a limitation that needs to be addressed for this cfDNA assay to be applied for the detection of residual disease. Despite the potential to have better sensitivity than personalized cfDNA analysis, mutations still could not be detected in all cfDNA samples from patients with relapse or metastasis. Therefore, if this cfDNA assay is applied for monitoring the residual disease, the results should be interpreted with caution, especially for patients with wild type RAS.

Among 31 patients with NED, genomic alterations in cfDNA were still detected in 12 patients, resulting in the low specificity when using this cfDNA assay to determine the presence or absence of disease. Ten of the 12 patients had been disease-free for more than 3 years, with the longest disease-free survival being 11 years. Moreover, no 2nd malignancy was detected among these patients. Although a longer follow-up is still needed to confirm the absence of relapse of the disease, the possibility of recurrence is very low, and the positive cfDNA analysis in these patients might not be interpreted as the presence of residual disease. Among 12 patients with a positive cfDNA test, the most common variant was a *TP53* mutation, which was detected in 66.7% of patients. *TP53* mutations were the most common genetic alterations in human cancers. A recent study demonstrated that low-frequency mutations in the *TP53* gene could be detected in cfDNA (21). These low-frequency *TP53* mutations were derived from normal tissue and progressively increased with age. By analyzing the association between age and the results of cfDNA analysis, our study also showed that positive cfDNA analysis was more common in patients who were 60 years or older. These results suggest that the genomic alterations detected in the NED group might be derived from clonal mutations in normal tissues instead of the residual colon cancer. By comparing the data from the UMD TP53 database, our result showed that 50% of the *TP53* mutations detected in the NED group were frequently or very frequently observed in human cancers. These cancer-like mutations derived from normal tissues contribute to the cfDNA in the plasma and bring challenges to the specificity of using commercial cfDNA assays in an adjuvant setting. Therefore, the results obtained using the commercial cfDNA assay alone without filtering out the clonal mutations derived from normal tissues should be interpreted with caution.

Molecular profiling and detecting the emergence of resistant mutations are also important applications of cfDNA (22). In the group of relapse or metastasis, *RAS* mutations, including 1 *KRAS* Q61H and 1 *NRAS* Q61H mutation, were detected in 2 of 16 patients with wild type RAS. Disease progression on anti-EGFR therapy developed in these 2 patients several months before cfDNA analysis, suggesting these mutations might be acquired resistant mutations. *BRAF* mutations and *HER2* amplification were two new therapeutic targets for mCRC (23, 24). This cfDNA assay could detect *ERBB2* amplification in the patient with *HER2*-positive CRC and *BRAF* mutations in *BRAF*-mutant CRC patients. Compared with mutational profiling routinely analyzed using tumor samples, *TP53, SMAD4, APC, AKT1, GNAS, IDH1, IDH2*, and *PDGFRA* mutations were the additional mutations identified by this cfDNA assay. Among these mutations, many small molecular inhibitors, including axitinib, dasatinib, imatinib, lenvatinib, nilotinib, nintedanib, and posatinib, were available for the inhibition of PDGFR-α (25). Recently, ivosidenib and enasidenib were approved by the FDA to treat acute myeloid leukemia with *IDH1* and *IDH2* mutations (26). However, the efficacy of targeting these mutations in CRC patients is seldom reported and still needs to be further studied.

The commercially available cfDNA assay, which simultaneously detects many different types of mutations across a wide range of genes, might have the potential to provide better sensitivity than patient-specific cfDNA analysis when used in an adjuvant setting of CRC. Determining how to filter out the cancer-like genomic alterations derived from normal tissues is a critical issue that needs to be addressed. Compared with mutational profiling routinely analyzed using tumor samples, this cfDNA assay could identify not only the known actionable mutations but also several potential therapeutic targets for CRC.

## Data Availability Statement

The datasets presented in this study can be found in online repositories. The names of the repository/repositories and accession number(s) can be found here: https://dataview.ncbi.nlm.nih.gov/, PRJNA656410.

## Ethics Statement

The studies involving human participants were reviewed and approved by Institutional Review Board of National Cheng Kung University Hospital. The patients/participants provided their written informed consent to participate in this study.

## Author Contributions

R-HC, M-RS, and Y-MY helped the conception and study design. R-HC, P-CL, and M-RS developed the method. R-HC, P-CL, S-HC, S-CL, P-CC, and B-WL helped the acquisition of data. P-CL, M-RS, and Y-MY analyzed the data. R-HC and Y-MY wrote the paper. All authors contributed to the article and approved the final submitted version.

## Conflict of Interest

The authors declare that the research was conducted in the absence of any commercial or financial relationships that could be construed as a potential conflict of interest.

## Supplementary Material

Supplementary Figure 1 Mutant allele frequency in the NED and relapse or metastasis group.

Supplementary Table 1 The amount of cfDNA input in the NED and relapse or metastasis group.

Supplementary Table 2 Genomic alterations in relapse or metastasis group.

Supplementary Table 3 Genomic alterations in the NED group.

Supplementary Table 4 Features of *TP53* mutations in the NED group.
