# Supplementary material for: Clinical Utility of a Cell-Free DNA Assay in Patients With Colorectal Cancer
Source: Front Oncol. 2021 Mar 19;11:589673. doi: 10.3389/fonc.2021.589673 (PMC8017343; doi:10.3389/fonc.2021.589673)
Supplement: Supplementary Table 1 — The amount of cfDNA input in the NED and relapse or metastasis group. [file Table_1.DOCX]

**Supplementary Table 1**. The clinical information and the amount of cfDNA input in the NED and relapse or metastasis group.

| NED group | | | | |  | Relapse or metastasis group | | | | |
| --- | --- | --- | --- | --- | --- | --- | --- | --- | --- | --- |
| No. | TNM | Stage | Adjuvant chemotherapy | cfDNA input (ng) |  | No. | Relapse or metastasis at initial diagnosis | Site of metastasis | Chemotherapy when cfDNA testing | cfDNA input (ng) |
| 39 | T3N2M0 | III | FOLFOX | 22.7 |  | 9 | Relapse | Lung, bone | Nil | 47.0 |
| 46 | T1N1M0 | III | FOLFOX | 18.2 |  | 14 | Relapse | Lung, LN | FOLFIRI + Bev | 26.0 |
| 36 | T3N1M0 | III | FOLFOX | 19.8 |  | 12 | Relapse | Liver, peritoneum | FOLFOX | 47.2 |
| 47 | T2N1M0 | III | XELOX | 22.2 |  | 7 | Metastasis | Lung, liver, LN | FOLFOX | 49.0 |
| 49 | T3N0M0 | II | 5FU/LV | 9.6 |  | 8 | Metastasis | Lung, liver, peritoneum | FOLFOXIRI + Bev | 46.4 |
| 54 | T3N1M0 | III | FOLFOX | 13.1 |  | 25 | Metastasis | Lung, liver, bone | FOLFIRI + Bev | 26.8 |
| 55 | T3N1M0 | III | FOLFOX | 12.3 |  | 26 | Metastasis | Lung, liver, LN | FOLFIRI + Bev | 21.1 |
| 56 | T3N2M0 | II | 5FU/LV | 11.3 |  | 28 | Relapse | Lung | Nil | 47.4 |
| 34 | T3N0M0 | III | FOLFOX | 26.6 |  | 19 | Relapse | Lung | FOLFIRI + Bev | 29.3 |
| 35 | T3N1M0 | III | FOLFOX | 20.5 |  | 2 | Metastasis | Lung | FOLFIRI + Bev | 50.0 |
| 57 | T1N1M0 | III | FOLFOX | 13.8 |  | 4 | Metastasis | Peritoneum | FOLFIRI + Bev | 20.3 |
| 59 | T3N2M0 | III | FOLFOX | 13.3 |  | 1 | Metastasis | Peritoneum, ovary | FOLFIRI + Bev | 23.6 |
| 40 | T3N1M0 | III | FOLFOX | 45.6 |  | 30 | Metastasis | Liver, LN | Cap + Bev | 10.5 |
| 37 | T3N2M0 | III | FOLFOX | 12.4 |  | 15 | Metastasis | Lung, liver, LN, peritoneum | FOLFOX | 45.2 |
| 33 | T3N1M0 | III | FOLFOX | 13.7 |  | 24 | Metastasis | Liver | Nil | 26.2 |
| 44 | T3N2M0 | III | FOLFOX | 18.2 |  | 13 | Relapse | Liver, bone | Nil | 9.0 |
| 51 | T4N1M0 | III | FOLFOX | 41.8 |  | 18 | Relapse | Peritoneum | Nil | 45.2 |
| 38 | T1N2M0 | III | FOLFOX | 42.6 |  | 21 | Metastasis | Lung | FOLFOX | 21.6 |
| 41 | T3N0M0 | II | 5FU/LV | 37.9 |  | 10 | Metastasis | Peritoneum | FOLFIRI + Bev | 49.0 |
| 52 | T3N0M1 | IV | FOLFIRI + Bev | 13.4 |  | 23 | Metastasis | Liver | Nil | 22.2 |
| 5 | T3N0M0 | II | 5FU/LV | 17.0 |  | 22 | Metastasis | Peritoneum | Nil | 11.8 |
| 60 | T3N0M0 | II | 5FU/LV | 10.0 |  | 3 | Metastasis | Lung | FOLFOX | 34.3 |
| 45 | T3N0M0 | II | 5FU/LV | 20.0 |  | 11 | Metastasis | Lung, LN | FOLFIRI + Cet | 47.0 |
| 48 | T3N2M0 | III | 5FU/LV | 15.0 |  | 16 | Metastasis | Peritoneum | Pembrolizumab | 14.2 |
| 32 | T3N2M0 | III | FOLFOX | 17.4 |  | 17 | Metastasis | Liver, LN, bone | FOLFOX + Bev | 50.0 |
| 31 | T2N1M0 | III | 5FU/LV | 40.6 |  | 20 | Metastasis | Liver, peritoneum | FOLFIRI + Bev | 5.1 |
| 43 | T3N1M0 | III | FOLFOX | 16.6 |  | 27 | Metastasis | Liver | FOLFOX + Bev | 43.7 |
| 42 | T3N2M0 | III | 5FU/LV | 20.4 |  | 29 | Relapse | Lung | Nil | 6.53 |
| 50 | T3N0M0 | II | 5FU/LV | 9.5 |  |  |  |  |  |  |
| 58 | T3N1M0 | III | 5FU/LV | 15.7 |  |  |  |  |  |  |
| 53 | T3N2M0 | III | FOLFOX | 22.0 |  |  |  |  |  |  |

Abbreviations: 5FU, 5-fluorouracil; Bev, bevacizumab; Cap, capecitabine; Cet, cetuximab; cfDNA, cell-free DNA; FOLFIRI, folinic acid, 5-fluorouracil, irinotecan; FOLFOX, folinic acid, 5-fluorouracil, oxaliplatin; FOLFOXIRI, folinic acid, 5-fluorouracil, oxaliplatin, irinotecan; LV, leucovorin; NED, no evidence of disease; XELOX, xeloda, oxaliplatin;
